# Apgar score or birthweight in Chihuahua dogs born by elective Caesarean section: which is the best predictor of the survival at 24 h after birth?

**DOI:** 10.1186/s13028-020-00538-y

**Published:** 2020-07-23

**Authors:** Jasmine Fusi, Massimo Faustini, Barbara Bolis, Maria Cristina Veronesi

**Affiliations:** Department of Veterinary Medicine, Università degli Studi di Milano, Via dell’Università, Lodi, 6-26900 Italy

**Keywords:** Apgar score, Birthweight, Chihuahua, Dog, Neonatal assistance, Outcome

## Abstract

**Background:**

In the dog, the correct management of parturition and the prompt neonatal evaluation and assistance can reduce the perinatal mortality rates that are particularly high in toy breeds. Newborn evaluation and factors addressing prognosis are pivotal to guarantee the correct neonatal assistance. Assessment of the Apgar score with viability classification and birthweight are recognized as predictors for neonatal survival in dogs, but breed-specific data are needed for a more feasible application in the dog species, in which wide differences among breeds are known. The present study aimed therefore to: (a) assess the role of Apgar score and birthweight as predictors for the survival of Chihuahua newborn puppies in the first 24 h of life; (b) to assess a cut-off of the Apgar score and birthweight values that can predict the survival of Chihuahua newborn puppies in the first 24 h after birth; (c) to assess the possible effect played by maternal parity, newborn gender and litter-size on Apgar score in Chihuahua newborn puppies, in order to provide breed-specific data for a better neonatal assistance.

**Results:**

Data obtained from 176 normal developed Chihuahua puppies born by elective Caesarean section, showed that 62%, 28% and 10% of puppies were classified in the Apgar score classes 7–10, 4–6 and 0–3, respectively, with survival at 24 h after birth of 97%, 96%, 39%, in the three Apgar classes of viability, respectively. Apgar score was a better predictor for survival at 24 h after birth than birthweight (AUC 0.93, P < 0.0001; AUC 0.69, P < 0.01, respectively). Litter-size of 7 puppies/litter plays a negative effect on Apgar score. Apgar score is a better predictor of survival at 24 h than birthweight, and the best cut-off of Apgar score for survival at 24 h after birth is 4, with 96% sensitivity and 77% specificity.

**Conclusions:**

The different proportion of “normal viable” and “less viable” neonates in comparison to other studies highlights that Chihuahua puppies born by elective Caesarean section should be carefully evaluated at birth to provide correct assistance.

## Background

In mammals, the process of birth represents a challenging time for the offspring, so that some losses can occur immediately before, during or after delivery, mainly due to asphyxia [1]. These losses can lead to significant perinatal mortality in canine species [2], up to 30%, representing an issue in canine reproduction and requiring further investigations and control [3]. The reasons underneath this phenomenon can be addressed to many factors. Among the specie-specific causes, the polytocous nature of the dog, in which multiple fetuses are delivered during an expulsion phase that can last for several hours, predisposes some fetuses to hypoxia, thus impairing their viability [4–6]. Furthermore, some canine breeds, such as toy and small-sized breeds [4, 7] are characterized by high mortality of newborns [4, 7], often in relation to the high predisposition for dystocia, putting in danger the fetal and neonatal viability [8].

A correct management of parturition [9], limiting the length of the expulsion phase, is therefore pivotal for providing the best chances to survive to the newborns [10, 11]. Elective timely planned Caesarean sections, performed using anesthetic protocols targeted to limit side effects on newborns, were reported to be associated to the best survival rate in canine newborns [11, 12].

Survival of newborns is also dependent on the recognition of viability class. Among the methods to assess neonatal viability immediately after birth, Apgar score received an extensive interest in human and veterinary medicine, and, in the last decade, also for the evaluation of viability of canine newborns [3, 6, 12–21]. Thanks to its demonstrated simplicity, the Apgar score provides a prompt classification of newborn distress allowing a more accurate neonatal assistance.

To improve the accuracy in classification and management of newborn viability, after the first Apgar scoring model proposed for puppies [13], a slight change was suggested for some brachycephalic breeds [16]. On the basis of their physiologic lower heart rate, indeed, the score was modified to assess possible breed-related necessities, improving the usefulness of the original model [16]. Recently, Vilar et al. [20] reported that French bulldogs born by Caesarean section need more clinical care than other breeds, with resuscitation procedures required in more than 50% of the French bulldog puppies. The proportion of puppies needing resuscitation was high also in Chihuahua puppies, 23%. Considering that Chihuahua dogs are at risk for dystocia, elective Caesarean sections are very often performed in this breed for the health of mothers and puppies. Although performed with surgical and anesthetic protocols aimed to minimize the side effects on newborns, a careful evaluation of newborn viability is pivotal.

To the authors’ knowledge, Chihuahua newborns do not need a targeted Apgar score, as it was done for French Bulldog by Vilar et al., [20], as the heart rate and the other parameters reported in literature [20, 22] are in agreement with the Apgar score introduced by Veronesi et al. [13].

In several studies [3, 6, 12, 13, 17, 19, 20] the cut-off of Apgar score to distinguish “normal viable” from “less viable” puppies, was 7, while Vassalo et al. [14] used a cut-off of 6 and Batista et al. [16] used a limit of 5. In most cases, the group of “less viable” newborns included puppies scored 0–3 and classified as “severely distressed” plus the newborns scored 4–6 and classified as “moderately distressed”, while Vilar et al. [20] considered only puppies scored < 5 as newborns with reduced vitality.

The Apgar score was also recognized to be a prognostic factor for the survival in the first 24–48 h of life [6, 12, 13, 16, 17, 20]. Survival rate was reported to range between 43 and 91% in puppies scored 0-3, and between 88 and 100% for those scored ≥ 4 [6]. Subsequently, Mila et al. [17] suggested a value of Apgar score of 6 as the best cut-off to predict neonatal survival in dogs, with a sensitivity of 70% and specificity of 87.5%. That study, performed on 346 newborn puppies belonging to different breeds, showed that 22% of puppies with Apgar score < 6 died within 24 h from birth.

It is necessary to highlight that, among many factors affecting newborn viability at birth, when newborns are delivered by elective Caesarean section, the anesthesia protocol used plays a pivotal role. Although a standardized protocol is not recognized, specific requisites for Caesarean section, such as minimal depression on cardio-respiratory system, total reversibility of side effects on neonates, and also long-lasting analgesia, quick and safe maternal awakening, normal maternal care of the offspring are required [23].

Beside the role of Apgar score as a predictor of newborn survival, also the birthweight and the weight gain were reported to be pivotal for the chance to survive of a newborn dog [5, 7, 9, 17, 24, 25]. Low birthweight was recognized as a negative prognostic factor for diseases or mortality of newborn puppies [5, 7, 9, 25], especially in toy and small-sized breeds [5], and some studies detected the threshold of bodyweight at birth to predict survival [21, 25]. Due to the wide range of body size and bodyweight among canine breeds, it is therefore important to provide reference data specific for breed or bodysize, for a more appropriate neonatal evaluation in dogs [21, 25]. Chihuahua is the smallest canine breed, in which the ideal bodyweight of an adult can range between 1.5 and 3 kg, with an accepted range of 0.5–3 kg (Federation Cynologique Internationale standard n.218 - 15/09/2010) [26]. Although the availability of reference data about birthweight and survival prognosis could be very useful, the authors are not aware of specific studies reporting data about Chihuahuas birthweight in relation to survival prognosis.

Therefore, the aims of the present study were: (a) to assess the role of Apgar score and birthweight as predictors for survival of Chihuahua newborn puppies at 24 h after birth; (b) to assess the cut-off of Apgar score and birthweight for the best rate of survival at 24 h of life in Chihuahua newborn puppies; (c) to assess the possible effect played by maternal parity, newborn gender and litter-size on Apgar score in Chihuahua newborn puppies, in order to provide breed-specific details for a better neonatal assistance.

## Methods

The study was performed in accordance with the ethical guidelines provided by the animal welfare committee and all the procedures were carried out according to the Italian legislation about animal care (DL 116, 27/01/1992) and to the European Guidelines on Animal Welfare (Directive 2010/63/EU). A written informed consent was signed by the owners, giving the permission to submit each dog to elective C-section and allowing the record of clinical data for research purposes.

### Animals

In this study 57 purebred Chihuahua bitches, aged from 2 to 5 years old, 24 primiparous and 33 multiparous, were enrolled. They were healthy, regularly vaccinated and dewormed, and showed a body condition score of 3/5 at the time of mating. The bitches were fed with a commercial diet, and the amount of food was modified during pregnancy according to the number of fetuses, in order to meet the metabolic and energetic needs.

All of them were fully monitored from the time of mating until the end of pregnancy. Because of the high risk of dystocia in Chihuahuas, for the health of bitches and puppies, an elective Caesarean section was planned for all the subjects enrolled in this study. The date of surgery was estimated, as previously reported [3, 19, 27, 28], by considering the value of blood plasma progesterone concentration at the sole mating plus the embryonic/fetal biometry. However, in the last week of pregnancy, a clinical and ultrasonographic supervision of mothers and fetuses was performed to verify maternal and fetal wellbeing and possible impending parturition. Blood progesterone concentration was also rechecked to verify the actual final stage of pregnancy, based on the finding of progesterone concentrations ≤ 2 ng/mL [19, 27].

### Elective Caesarean section and newborn evaluation

Elective Caesarean section was performed in all the cases with the same anesthetic protocol and surgical procedures, as previously reported [19, 27], aimed to minimize the negative impact on the viability of newborns. Briefly, a first injection of metoclopramide (0.2 mg/kg s.c.) and cefazolin (25 mg/kg i.v.) was done, while a support with an oxygen mask was provided. Then, with a propofol i.v. infusion (4 mg/kg), anesthetic induction was obtained. Lidocaine infiltration on the site of the surgical incision (2 mg/kg) was performed. The protocol was followed by maintenance in isoflurane and oxygen. Caesarean section was performed with a ventral midline laparotomy. Only after the extraction of the last fetus, the bitched were administered tramadol (3 mg/kg i.v.) and oxytocin (0.15 IU/kg i.m.), as previously reported [19, 27].

The time lapse between induction in anesthesia and the extraction of the last fetus was recorded. Immediately after uterine extraction, two expert neonatologists provided assistance to the newborns. Within a maximum of 5 min after extraction, each puppy was submitted to a single evaluation of viability with the Apgar score reported by Veronesi et al. [13]. In summary, each puppy was evaluated for mucus color, heart rate, reflex of irritability, motility and respiratory efforts with a score from 0 to 2 for each parameter, where 0 represents the worst score and 2 represents the optimal condition, for a final score ranging between 0 and 10 [13]. According to that, each puppy was assigned to one of the three classes of newborn viability: absence of newborn distress for Apgar score 7–10, moderate distress for Apgar score 4–6 and severe distress for Apgar score 0–3. Puppies scored as 7–10 were submitted to the routine neonatal cares, while those scored < 7 [13, 14] were submitted to a different degree of neonatal assistance or resuscitation according to their degree of viability [13, 29]. To complete the neonatal evaluation, after the identification of each newborn with colored collars, the absence of gross malformations was checked, and newborn gender and birthweight recorded. Birthweight was measured before nursing, with an electronic scale, calibrated in 1 g increments.

In all cases, bitches and litters were discharged when the mothers were awake and demonstrated normal maternal behavior with puppies, and after assessing that the mammary secretions were available to the newborns. If mammary secretions were not available, newborns were fed with a commercial milk replacer formula (Puppy Pro-Tech, Royal Canin ®) at the dose of 3 mL/100 g of bodyweight every 2 h.

As previously reported [13], newborn outcome (alive/dead) was checked 24 h after birth.

### Statistical analysis

Among several factors, the short-term survival of newborn dogs is influenced by neonatal viability (Apgar score) and birthweight, thus the first statistical analysis was done to ascertain the role of the Apgar score and of the birthweight on the survival of puppies at 24 h after birth (alive/dead) by Kruskall-Wallis test, followed by binomial logistic regression. In a second time, for the parameter(s) significantly associated to newborn outcome at 24 h after birth (alive/dead), a ROC curve was calculated to assess the prognostic value of the two parameters tested. ROC analysis was performed by consulting the website Easyroc (http://www.biosoft.hacettepe.edu.tr/easyROC/). Afterwards, a cut-off for newborn outcome at 24 h after birth (alive/dead) was detected for both Apgar score and birthweight by MaxKappa test, providing sensitivity, specificity, positive predictive value and negative predictive value. Furthermore, an ANOVA test was used to assess the possible effect played on Apgar score by maternal parity (primiparous/multiparous), newborn gender and litter size. For litter size a post hoc Tuckey test was applied to detect possible differences among Apgar score values according to the number of puppies in the litters.

Statistical significance was set for P < 0.05 (JASP®,ver 9 for Windows platform).

## Results

### Clinical findings

From the 57 bitches enrolled, a total of 179 Chihuahua puppies were born via elective Caesarean section, with litter size ranging from 1 to 7 (mean ± SD: 4.1 ± 1.39). The mean (± SD) interval of time between induction in anesthesia and the extraction of the last fetus was 18.7 ± 8.3 min.

Of the 179 puppies 3 neonates, belonging to different litters, were euthanized because of severe cleft palate, so that data were collected from 176 newborns, 80 males (45%) and 96 females (55%), 75 born from primiparous (43%) and 101 born from pluriparous (57%) bitches. One-hundred and sixty (91%) puppies were alive 24 h after birth, while 16 (9%) died within the first 24 h after birth.

### Apgar classification and survival

Data about the distribution of the Apgar class of viability in the 176 Chihuahua newborn puppies are reported in Table 1.

**Table 1:** Distribution of Apgar viability classes in the 176 Chihuahua newborn puppies

	Apgar score 7–10 N puppies (%)	Apgar score 4–6 N puppies (%)	Apgar score 0–3 N puppies (%)
Total N = 176	109 (62%)	49 (28%)	18 (10%)

Data about the outcome of the newborns at 24 h after birth (alive/dead), according to the distribution of the Apgar class of viability in the 176 Chihuahua newborn puppies, are reported in Table 2.

**Table 2:** Newborns outcome at 24 h after birth (alive/dead) according to the Apgar class in the 176 Chihuahua puppies

	Apgar score 7–10 N puppies (%)	Apgar score 4–6 N puppies (%)	Apgar score 0–3 N puppies (%)
Alive 24 h after birth	106/109 (97%)	47/49 (96%)	7/18 (39%)
Dead 24 h after birth	3/109 (3%)	2/49 (4%)	11/18 (61%)

### Birthweight and survival

Birthweight, expressed as mean ± SD and the range of weight in the 160 alive Chihuahua puppies and in the 16 dead puppies at 24 h after birth was: 141 ± 34.3 g (70–270 g) and 107 ± 49.8 g (60–180 g), respectively.

### Apgar score and birthweight AUCs and cut-off

The first statistical analysis by Kruskal–Wallis test showed that both Apgar score (P < 0.01) and birthweight (P < 0.05) were significantly associated with the outcome of the newborn (survival or death within 24 h after birth). The subsequent logistic regression, however, showed that only Apgar score was significantly related to the outcome of the newborn (P < 0.001), while birthweight was not significantly related to the outcome at 24 h after birth (P < 0.1).

**Fig. 1:**
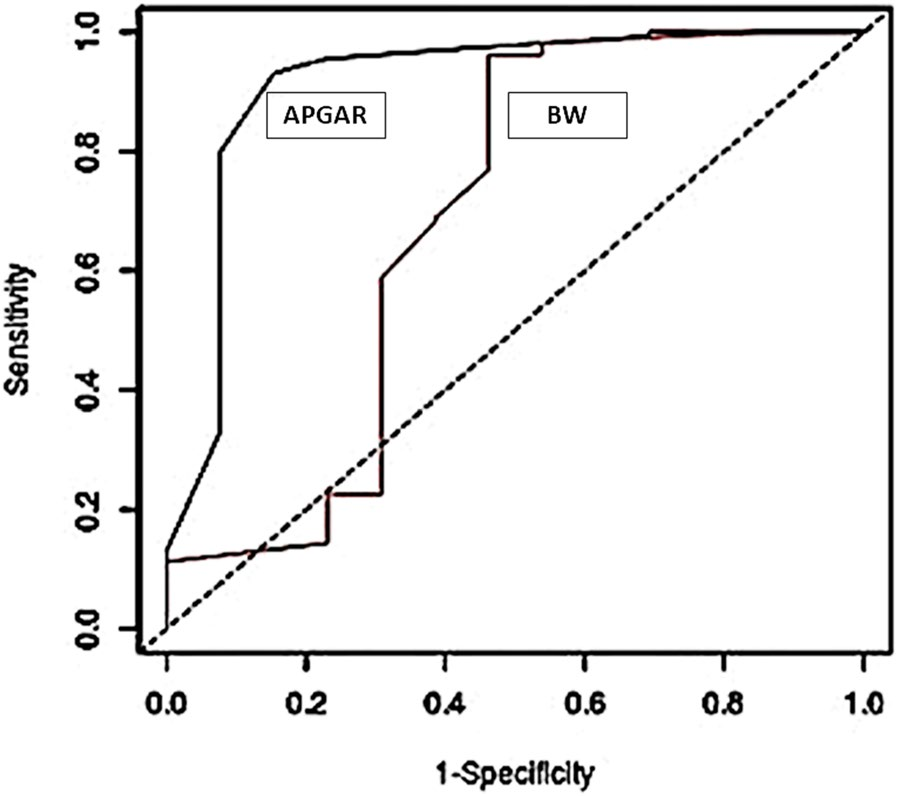
Comparison between AUCs related to Apgar score and birthweight (BW). Data are obtained from the evaluation of the 176 Chihuahua dog newborn puppies enrolled in the present study

The comparison of the ROC curves for Apgar score and for birthweight, showed that in both cases the area under curve (AUC) was statistically significant (Apgar score: AUC 0.93, P < 0.0001; birthweight: AUC 0.69, P < 0.01), with marked differences between the two curves (Fig. 1) and with a better feasibility of the ROC curve for Apgar score.

Afterwards, a MaxKappa test was applied to detect the value of the cut-off predicting the outcome at 24 h after birth for both Apgar score and birthweight, showing that the best cut-off value for Apgar score was 4, whilst the value identified for birthweight was 80 g (Table 3).

**Table 3:** Results of the MaxKappa test

	Apgar score cut-off 4			Birthweight cut-off 80 g		
	Value	Lower limit	Upper limit	Value	Lower limit	Upper limit
Sensitivity	0.956	0.912	0.982	0.981	0.946	0.996
Specificity	0.769	0.462	0.950	0.462	0.192	0.749
Positive predictive value	0.981	0.929	0.992	0.957	0.862	0.991
Negative predictive value	0.588	0.404	0.890	0.667	0.402	0.874

Data from MaxKappa test to detect the cut-off value for newborn outcome for both Apgar score (cut-off: 4) and birthweight (cut-off: 80 g) in the 176 Chihuahua newborn puppies enrolled in the study

### Apgar score and effect of maternal parity, newborn gender and litter size

Results about the possible effect played by maternal parity, newborn gender and the litter-size on Apgar score showed that only the litter size played a significant effect (P < 0.05). The subsequent Tuckey post hoc test detected significantly lower Apgar score values when the litter size was equal to 7 than in litters with 2 to 6 fetuses (Table 4).

**Table 4:** Apgar score values in relation to different parameters

	Apgar score (mean + SD)
Maternal parity	
Primiparous (n = 75)	6.59 ± 1.90
Multiparous (n = 101)	6.43 ± 1.98
Newborn gender	
Males (n = 80)	6.76 ± 1.96
Females (n = 96)	6.33 ± 1.88
Litter-size	
1 (n = 5)	5.2 ± 2.28^ab^
2 (n = 17)	6.82 ± 1.98^a^
3 (n = 35)	6.14 ± 1.99^a^
4 (n = 53)	6.87 ± 1.87^a^
5 (n = 35)	6.46 ± 1.80^a^
6 (n = 24)	7.08 ± 1.59^a^
7 (n = 7)	4.71 ± 2.14^b^

Factors considered are: maternal parity (primiparous/multiparous), newborn gender (male/female) and litter-size. Data shown are from the 176 Chihuahua newborn puppies enrolled in the study

^a, b^Within column denote statistical significance with P < 0.05

## Discussion

The present study aimed to investigate the role of Apgar score and of birthweight as predictors for the survival of Chihuahua newborn puppies born by elective Caesarean section in the first 24 h of life, and to define the cut-off for Apgar score and birthweight values prognosing the survival in the first 24 h. Other than that, the study aimed to assess the possible effect played by maternal parity, newborn gender and litter size on Apgar score.

On a total of 179 puppies, 1.7% showed severe anatomical defects and were euthanized. Although the actual incidence is understudied, this percentage is a bit lower than the 2.8% of cleft palate previously reported [30], and fit with the range of malformations of 1–3% reported by other studies [20, 31]. Excluded those 3 malformed puppies, 160/176 (90%) of Chihuahua puppies born by elective Caesarean section were alive at 24 h after birth, considered as the most challenging timespan for the newborn dogs [19]. This percentage is in agreement with the reported 86–92% [3, 13, 19] and slightly lower than the reported 95–97% [14, 17, 18, 21]. It is necessary to note that data from the cited studies were drawn from several canine breeds and from puppies born by different types of parturition (spontaneous vaginal delivery, manually assisted parturition, emergency or elective Caesarean section), and, in case of Caesarean section, also using different anesthetic protocols. Although all factors could affect the newborn viability and survival [4, 7, 11, 12], the most important is represented by the different anesthetic protocols, especially when elective Caesarean section is concerned. It is therefore to note that, in the present study, all puppies born by elective Caesarean section performed with the same anesthetic protocol, using propofol induction followed by isoflurane maintenance, demonstrated to have positive effects on newborn survival at 7 days of age [32].

According to the distribution of the Apgar classes of viability, 62% of the 176 normal developed newborns were considered as “normal viable” puppies (Apgar score 7–10), 28% as “moderately distressed” puppies (Apgar score 4–6), and 10% as “severely distressed” puppies (0–3). Comparing the results of this study with the bibliography is difficult, also considering the wide variability of study designs (different breeds enrolled, different type of parturitions, and especially different anesthetic protocols for Caesarean section). However, it is to note the lower proportion of “normal viable” newborns than the 85–96% reported in some studies by [3, 13, 19, 33], while it is higher than the 51% reported for puppies born by dystocia terminated by emergency Caesarean section or vaginal delivery [18], and similar to the 48–64% reported for puppies born by elective Caesarean section [20]. The percentage of 28% referred to the “moderately distressed” puppies is, in turn, higher than the 2–10% reported by [3, 13, 18], but more similar to the 34–44% reported by Vilar et al. [20]. The 10% proportion of “severely distressed” puppies, is higher than the reported 2–4% [3, 13], more similar to the 8% reported by [20, 33], but markedly lower than then the 43% reported by Titkova and colleagues [18]. Data from the present study showed, therefore, that in Chihuahua dogs born by elective Caesarean section about one-third of the newborns are classified as “less viable” (Apgar score 0–6), despite the timely performed surgery, anesthetic protocol used aimed to minimize side-effects on newborns and bitches, and the prompt evaluation and assistance performed by an experienced neonatologist.

The outcome of the newborns at 24 h of life (alive/dead) according to the distribution of viability classes based on Apgar score showed that 97% of puppies scored 7–10, 96% of puppies scored 4–6 and 39% of puppies scored 0–3 were alive 24 h after birth. When compared to previous studies, the survival rate in puppies scored as 7–10 is in agreement with the 95–100% reported before [3, 13, 18–20, 33]. Similarly, the survival rate in puppies scored as 4–6 agrees with the 94–100% previously reported [18, 20]. The survival rate of puppies scored 0–3, 39%, is in agreement with the reported 40–100% [18, 20], but differed from the 0% reported by others [3, 19].

When birthweight and outcome of the newborns at 24 h after birth were considered, the birthweight range in surviving Chihuahua puppies (70-270 g) was very similar to the birthweight range (70–250 g) reported by [3] in 50 small-sized normal surviving puppies and in agreement with the median of 134 g reported for toy breeds by Tesi et al. [21]. When compared with birthweight of Chihuahua newborns, moreover, it was in agreement with the mean ± SD of 119.6 ± 25.6 g [25] and with the range of 80–276 g reported for small-sized-breed puppies [17]. Although low birthweight was recognized as a negative prognostic factor also in newborn dogs [7, 25], few studies provided the exact indications about what should be considered as “low” birthweight, in dependence on several factors, such as body-size or even breed [21, 25]. Mugnier et al. [25] reported the birthweights from 6694 newborn puppies of different breeds and suggested a breed-specific approach to identify at-risk puppies. For Chihuahua newborns, the authors reported quartile values ranging between 102 g for the first quartile and 133 g for the third quartile.

Thus, recognized the role of Apgar score and birthweight on survival of canine newborns, and aware of the need for breed-specific data, the main purpose of the present study was to assess which of the two was the best predictor of survival at 24 h after birth, and to establish a cut-off value for both of the two parameters for predicting the newborn outcome at 24 h after birth in Chihuahua newborn dogs. The regression analysis and following ROC curves, demonstrated the higher power of Apgar score (AUC 0.93) than birthweight (AUC 0.69) as a predictor for the survival at 24 h after birth in Chihuahua newborn dogs. Furthermore, the detection of the cut-off values for the newborn outcome at 24 h, showed that the Apgar score cut-off 4 was a better predictor for newborn outcome in comparison to the birthweight cut-off of 80 g, in term of sensitivity, specificity, positive and negative predictive value. Mila et al. [17] reported the Apgar score cut-off in a large population of puppies belonging to many breeds and identified an Apgar score cut-off equal to 6, with a 70% sensitivity and 87.5% specificity, whilst in the present study the Apgar score cut-off had 96% sensitivity and 77% specificity. It has to be observed that in that study the Apgar score was measured within 6 h after birth, and not immediately after birth, as it was done in the present study, so that most of the puppies could have already improved their viability. Beside the role of Apgar score, also birthweight [17, 24, 25] and weight gain [7, 24] are prognostic factors in newborn dogs. Considering birthweight, in a study on multiple breeds by Mugnier et al. [25] the authors reported that the cut-off value for Chihuahua puppies that should be considered is > 102 g. This value is markedly different from the cut-off value of 80 g found in the present study, and was characterized by 98% sensitivity and only 46% specificity. This difference, probably attributable to the different genetic characteristics of Chihuahuas studied in the present study from the population investigated by Mugnier et al. [25], once again, highlights the need for further investigations with a breed-specific and maybe, also, “colony”-specific approach.

A last comment concerns the effect of litter-size on Apgar score. In the present study, a mean of 4.1 litter size, with a range of 1–7, was found. The average number of puppies/litter is a bit higher than 3.1–3.6 puppies/litter reported for miniature/small/toy canine breeds [3, 4, 34]. In the present study, a significant reduction of Apgar score was found for litters of 7 puppies in comparison to all the smaller litters, with an exception for litters composed by only one puppy. This result indicates that, also when elective Caesarean sections are performed, increased litter sizes or litter size extremes can raise the risk for perinatal mortality in toy breeds, as previously reported [4, 5]. About the negative effect of litters composed of 7 puppies on Apgar score, it is possible to speculate that the intrauterine competition of this amount of fetuses can be an issue for the correct placentation, resulting in turn in a lower neonatal viability.

## Conclusions

The data obtained from the 176 Chihuahua newborn dogs enrolled in this study once again confirm the valuable role of Apgar score for the assessment and classification of the viability of newborn dogs. Moreover, the results showed that Apgar score is a better predictor for the outcome in the first 24 h after birth than birthweight, and that in Chihuahua newborns the best Apgar score cut-off for the outcome at 24 h after birth is 4. The different proportion of “normal viable” and “less viable” neonates in comparison to most studies highlights that Chihuahua puppies born by elective Caesarean section should be carefully evaluated at birth to provide the correct assistance. Lastly, litter-size of seven puppies/litter could negatively affect the neonatal Apgar score.

## Data Availability

The datasets used and/or analysed during the current study are available from the corresponding author on reasonable request.
